# Inertial Pocket Navigation System: Unaided 3D Positioning

**DOI:** 10.3390/s150409156

**Published:** 2015-04-17

**Authors:** Estefania Munoz Diaz

**Affiliations:** German Aerospace Center (DLR), Institute of Communications and Navigation, Oberpfaffenhofen, 82234 Wessling, Germany; E-Mail: Estefania.Munoz@dlr.de; Tel.: +49-8153-28-4132; Fax: +49-8153-28-1871

**Keywords:** step length, step detector, attitude, pedestrian, dead reckoning, orientation, pitch, vertical displacement, activity

## Abstract

Inertial navigation systems use dead-reckoning to estimate the pedestrian's position. There are two types of pedestrian dead-reckoning, the strapdown algorithm and the step-and-heading approach. Unlike the strapdown algorithm, which consists of the double integration of the three orthogonal accelerometer readings, the step-and-heading approach lacks the vertical displacement estimation. We propose the first step-and-heading approach based on unaided inertial data solving 3D positioning. We present a step detector for steps up and down and a novel vertical displacement estimator. Our navigation system uses the sensor introduced in the front pocket of the trousers, a likely location of a smartphone. The proposed algorithms are based on the opening angle of the leg or pitch angle. We analyzed our step detector and compared it with the state-of-the-art, as well as our already proposed step length estimator. Lastly, we assessed our vertical displacement estimator in a real-world scenario. We found that our algorithms outperform the literature step and heading algorithms and solve 3D positioning using unaided inertial data. Additionally, we found that with the pitch angle, five activities are distinguishable: standing, sitting, walking, walking up stairs and walking down stairs. This information complements the pedestrian location and is of interest for applications, such as elderly care.

## 1. Introduction

The market for positioning applications has been growing in recent years for both mass market and professional users. So far, most positioning applications are based on Global Navigation Satellite Systems (GNSSs), in particular the American global positioning system. However, due to signal blockage and strong multipath, the availability of GNSSs is degraded in certain scenarios, such as urban canyons, underground or indoors. The inertial navigation is an appropriate self-contained system to complement the GNSS in these challenging scenarios.

The inertial sensors, *i.e.*, accelerometer and gyroscope, are applied widely for pedestrian navigation and are continuously investigated due to the emergence of micro-electromechanical sensors (MEMS). Thanks to this technology, it is possible to embed size-reduced and low-cost inertial sensors in smartphones.

Inertial navigation systems use pedestrian dead-reckoning (PDR) to sequentially estimate the pedestrian's position. There are two types of PDR, the strapdown algorithm and the step-and-heading approach. The strapdown algorithm consists of the double integration of the accelerometer readings. The accumulated error in the position due to the integration of current MEMS inertial sensor readings is prohibitively high. Therefore, the strapdown algorithm is so far only feasible using strong corrections, such as zero velocity updates (ZUPT), thus for foot-mounted sensors.

This article describes algorithms for an inertial pocket navigation system. The sensor is located in the front pocket of the pedestrian's trousers. For the pocket location, the use of the step-and-heading approach is most appropriate. The step-and-heading approach estimates sequentially the pedestrian's position based on the previous position for each detected step, the step length and the heading:

(1) $$\begin{array}{c} X_{k}=X_{k-1}+S_{k}\cdot \cos\left(\psi_{k}\right)\\ Y_{k}=Y_{k-1}+S_{k}\cdot \sin\left(\psi_{k}\right) \end{array}$$

where *X* and *Y* represent the position in the *x*- and *y*-axis, *S* stands for the step length and *ψ* is the pedestrian's heading.

The step-and-heading approach, as Equation (1) reveals, consists of the estimation of the heading and the estimation of the step length. Even if only the heading is required, estimating the complete orientation is of high interest. The orientation is defined as the attitude angles roll (*ϕ*) and pitch (*θ*) and the heading angle yaw (*ψ*). The complete orientation is of interest, among other reasons, because the attitude angles can be used for the step length estimation part, although the vast majority of the literature algorithms use only inertial raw measurements to this end.

This article is focused on the step length estimation part. Every step should be detected prior to estimating the step length. The step length is defined as the distance measured in the heading's direction between two consecutive hits on the floor of the same foot. A stride length is the distance measured in the heading's direction between both feet. Therefore, the step length is twice the stride length, since the walk of a standard pedestrian is considered symmetric between both legs.

For the step-and-heading approach, there are different step detectors and step length estimators in the literature that will be analyzed in this article. However, as Equation (1) shows, there is still an important and unsolved aspect so far: the displacement in the vertical direction or the *z*-position.

This article aims at presenting the, from our knowledge, first step-and-heading approach based on unaided inertial data solving 3D positioning. Previous works in the area have introduced the pedestrian's vertical displacement by means of extra sensors, such as barometer, GNSS, WiFi access points, *etc.*, or extra information, like maps. The present work adds great value to the state-of-the-art because it uses only inertial data.

In Section 2, the most relevant related work in this area is gathered. Section 3 offers an overview of the pocket navigation system. Section 4 explains the detection of steps for horizontal surfaces and for steps up and down stairs. Additionally, the identification of five basic physical activities, *i.e.*, standing, sitting, walking, walking up stairs and walking down stairs, based on the pitch angle is detailed. Section 5 extends to different walking speeds the step length estimator already presented by the authors and suggests a calibration procedure for the proposed model. Section 6 details the vertical displacement estimator and the experimental set up that has been carried out in order to deduce a model based on the pitch angle. Section 7 presents the experimental work carried out to assess the step detector, step length estimator and the vertical displacement estimator. Finally, the conclusions and outlook are drawn in Sections 8 and 9, respectively.

## 2. Related Work

There is a large amount of work in the area of PDR. In [1], a classification of different types of PDR and a comparative study between algorithms proposed in the state-of-the-art can be found.

Particularly for the step-and-heading approach, a well-known approach to identify steps is to detect changes in the vertical displacement of the pelvis. This idea has been developed in [2] with the sensor attached to the belt. For other locations where the sensor is not close to the center of mass of the pedestrian, like the pocket, or completely decoupled from the pedestrian's motion, such as hand held or wrist worn, detecting changes in the vertical displacement of the pelvis does not offer optimal results.

Another step detector algorithm in the literature uses the acceleration signal. The simplest approach is finding peaks in the variance of acceleration in the z-axis of the navigation frame [3]. The peaks correspond to the step occurrences, because they are generated by the vertical impact when the foot hits the floor. This approach requires pre- and post-processing of the acceleration signal and a threshold to avoid spurious detections from small peaks. A similar approach identifies peaks in the magnitude of the complete acceleration [4–7]. The detection of these peaks requires also the use of thresholds to avoid spurious detections.

A common disadvantage of the approaches based on acceleration is that the pattern of the acceleration signal is greatly affected by the pedestrian's walking speed. Therefore, the determination of the thresholds for reliable step detection is challenging.

In [8], a step detector based on the pitch signal of a sensor mounted on the waist is presented. The Fourier analysis of the fundamental frequency of the pitch is proposed to detect the pedestrian's steps.

In [9], we presented a step detector also based on the pitch angle. This detector is optimized for a sensor placed in the pocket of the trousers or attached at any point of the leg and will be described for completeness and used in this article. The two advantages of our detector with respect to the state-of-the-art are that the detection is less complex, because it does not require a Fourier analysis of the pitch signal, and easier, because the pitch does not contain spurious peaks, like the acceleration signal. The threshold is fixed for all walking speeds, and the false detection and undetected steps rates are lower than the state-of-the-art detectors.

For the special case of the sensor held in the hand while the pedestrian's arm is swinging, the pitch angle for detecting steps offers excellent results. Additionally, the step detection can be directly done with the raw turn rate signals.

In [10], a classification of the smartphone motion modes between symmetrical and asymmetrical is proposed. For the symmetrical modes, such as texting and phoning, the more adequate step detector uses the acceleration signal. For the asymmetrical modes, such as swinging and front and back pocket of the trousers, the use of the pitch is recommended.

In [9], the maximum of the pitch signal is detected, and [10] uses the zero-crossing for the identification of steps. In [11], the zero-crossing of the pitch signal is also used to detect steps for a rigid ankle-mounted sensor.

There is still a common problem for all step detectors, which is the false positive rejection. This happens, for example, if the pedestrian is tapping on the smartphone and not actually moving. The peaks in the acceleration due to the tapping may be confused with the peaks produced when walking. In [10], a method is proposed to determine whether a step has actually been taken or not.

Regarding the step length estimation, the main current approaches can be classified depending on the location of the sensor, as specified in the comparative studies [1,12,13].

If the sensor is attached to the body near the center of mass, a classic approach is to model the human body as an inverted pendulum. The major constraint of this biomechanical model is the assumption of a kneeless biped. The model needs a previous calibration to determine the scaling parameter *K*. Additionally, the length of the pedestrians leg, *L*, is included in the model:

(2) $$S=K\cdot \sqrt{2Lh-h^{2}}$$

where *S* represents the estimated step length and *h* the vertical displacement of the pelvis for each step. This method has been applied in [14]. In [12], a more complex model based on two pendulums is analyzed. This model does not require previous calibration.

Another approach to estimate the step length makes use of the empirical relationship of the acceleration measured in the z-axis of the navigation frame and the step length [2,4]. This relationship was proposed by Weinberg in [15].

(3) $$S=K\cdot \sqrt[{4}]{a_{\max}-a_{\min}}$$

where *S* represents the estimated step length, *K* is a scaling parameter and *a*_max_ and *a*_min_ are the maximum and minimum values of the acceleration measured in the z-axis of the navigation frame for each step.

Therefore, the Weinberg approach requires a previous calibration to determine the scaling parameter *K*, and the estimated step length offers similar results compared to the model of Equation (2). However, the Weinberg approach's advantage is that it uses raw data and does not require recursively estimating the parameter *h*, whose computation may contain errors.

If there are no restrictions on the sensor placement [5,6,16,17], a widespread approach uses the relationship between step length and step frequency. This method also requires previous calibration to fit the scaling parameter. A more sophisticated model includes also the height of the pedestrian.

In [9], we presented a step length estimator based on the relationship between the pitch angle and the step length, which will be further described for completeness and used in this article. Our estimator is optimized for a sensor placed in the pocket of the trousers or attached at any point of the leg.

Regarding the vertical displacement estimation of the pedestrian for the step-and-heading approach, the barometer or altimeter is the most used sensor [18] to aid inertial systems. The barometer measures the atmospheric pressure. The altimeter is essentially the same instrument, but it matches the atmospheric pressure to the corresponding altitude. Both sensors, however, suffer from inherent dynamic influential factors, such as temperature and environmental pressure.

The satellite-based systems offer complete 3D positioning; thus, a GNSS-aided inertial step-and-heading approach could be a viable option. However, as mentioned in the Introduction, the signal blockage and strong multipath in indoor environments drastically reduces the availability of satellite-based positioning systems.

Having maps could help determining the *z*-axis position of the pedestrian making use of the floor number, which could be recognized if the building layout is different between floors. Additionally, 3D positioning is also possible with WiFi or ultra wideband points if a database or map of their position in the building is available. However, maps are not always available.

If the strapdown algorithm is used, the double integration of the *z*-axis acceleration yields the *z*-axis position of the pedestrian [19]. Future accurate sensors will allow the strapdown algorithm to be applied if the sensor is located on an arbitrary part of the pedestrian's body. With the current MEMS technology, the strapdown algorithm can only be applied if strong corrections, like ZUPT, are possible, and this, as previously mentioned, is only valid for foot-mounted sensors.

Therefore, it can be concluded that the state-of-the-art step-and-heading approach is able to offer 2D positioning. The z-axis position, or the pedestrian vertical displacement estimation, can only be offered by aiding the inertial sensors with extra sensors, such as a barometer, GNSS, WiFi access points, among others, or by having extra information, such as maps. This article presents the, from our knowledge, first unaided inertial step and heading navigation system solving 3D positioning.

## 3. System Overview

**Figure 1:**
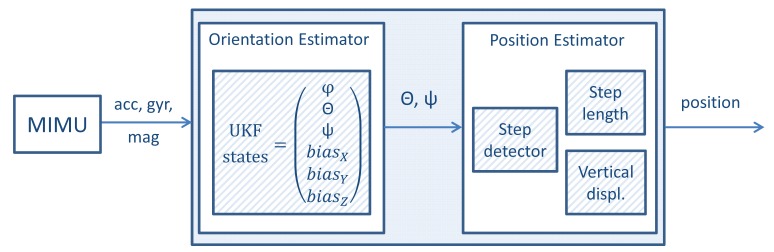
Overview of the structure of the inertial pocket navigation system.

The pocket navigation system is divided into two subsystems, hardware and software. The hardware subsystem, the left box in Figure 1, consists of a magnetic and inertial measurement unit (MIMU). All of the experiments and the data provided in this article have been recorded with the MIMU Xsens MTw.

The navigation system described in this article offers the advantage that the MIMU can be located in the front pocket of the trousers. No additional fastening is required unless the trousers are loose enough that the movement of the MIMU in the pocket is completely decoupled from the movement of the pedestrian's leg. For these cases, the sensor can be directly attached to the leg.

The software subsystem refers to the source code programmed in a laptop/tablet, which is represented in Figure 1 with the outer right box. It has two main parts, the orientation estimation algorithm and the position estimation algorithm. The inputs of the software subsystem are measurements of accelerometers, gyroscopes and magnetometers at a rate of 100 Hz, and the output is the pedestrian's position.

The orientation estimator is the first algorithm of the software subsystem. It consists of an unscented Kalman filter (UKF), whose states are the Euler angles roll, pitch and yaw and the biases of the gyroscope. The UKF prediction stage integrates the turn rate measurements and applies an auto-regressive model for the biases. The UKF update stage makes use of the accelerometer and magnetometer measurements to correct the orientation estimation. In [20], a detailed explanation of the orientation estimation is given.

The estimations of the pitch and yaw angles are transferred at a rate of 100 Hz to the position estimator, as represented in Figure 1. The position estimator consists of three algorithms: the step detector, the step length estimator and the vertical displacement estimator.

Unlike the orientation estimator, which follows a probabilistic approach using a UKF, the position estimator is deterministic. Thus, the uncertainty of the position *x, y* and *z* is not taken into account. In fact, the position uncertainty always increases with time due to the lack of additional information to correct the position estimation.

The pocket navigation system uses the pitch angle to detect steps in horizontal surfaces, as well as steps up and down in stairs. Additionally, five basic physical activities can also be identified with the pitch angle, and this angle is used to estimate the step length and the vertical displacement of the pedestrian. In the following sections, the three main algorithms of the position estimator, *i.e.*, step detector, step length estimator and vertical displacement estimator, will be detailed.

## 4. Step Detector

This section details the proposed step detector based on the pitch angle. In [9], the authors presented this step detector for horizontal surfaces, which will be shortly presented for completeness. Then, the proposed step detector is broadened to stairs adding a great value to the state-of-the-art, because stairs are a challenging scenario where the literature step detectors have trouble detecting steps up and down. Additionally, the identification of five basic physical activities based on the pitch angle will be proposed. The physical activity complements the location and is of interest for applications, such as elderly care.

### 4.1. Horizontal Surfaces

If the inertial sensor is introduced in the pocket of the trousers, the estimated pitch angle describes the opening angle of the pedestrian's leg. Knowing how the leg moves is valuable information.

**Figure 2:**
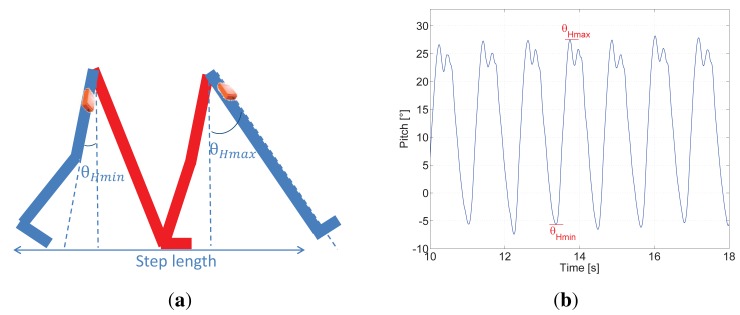
Pitch angle representation in schema and estimated signal. (**a**) Representation of the legs during a step. A step is completed every time the leg which has the sensor, in this case the blue one, hits the floor; (**b**) The blue line represents the pitch angle estimation during a walk. In this figure, seven steps are represented.

Figure 2b shows the estimation of the pitch angle during a walk for seven steps. The maximum elongation of the leg, when the foot is still in the air, is indicated as *θ*_Hmax_, and the second positive peak occurs as a consequence of the foot hitting the floor. The lowest negative angle is indicated as *θ*_Hmin_. Figure 2a shows that *θ*_Hmax_ is much larger than *θ*_Hmin_. The reason for this difference is that the pedestrian bends the knee of the rear leg considerably when walking.

The vertical dashed line represents a pitch angle equal to zero. Therefore, if the pedestrian is standing, the legs are closed and the pitch angle is zero.

The amplitude of the opening angle of the leg or pitch amplitude is defined as:

(4) $$\Delta \theta_{\text{H}}=\theta_{\text{Hmax}}-\theta_{\text{Hmin}}.$$

Our step detector consists of the identification of the maximum of the pitch angle, because each maximum indicated as *θ*_Hmax_ corresponds to a step. As Figure 2 shows, the pitch angle is ideal for detecting steps, because its cyclic nature during the walk makes the maximum or the minimum occur clearly only once per step. No varying thresholds with the pedestrian speed or post-processing of the pitch signal are needed.

### 4.2. Stairs

This section aims at explaining the extension of the detection of steps in horizontal surfaces to steps up and down in stairs. The detection of steps in stairs will be explained through the study of the position and movement of the legs when walking up and down stairs.

**Figure 3:**
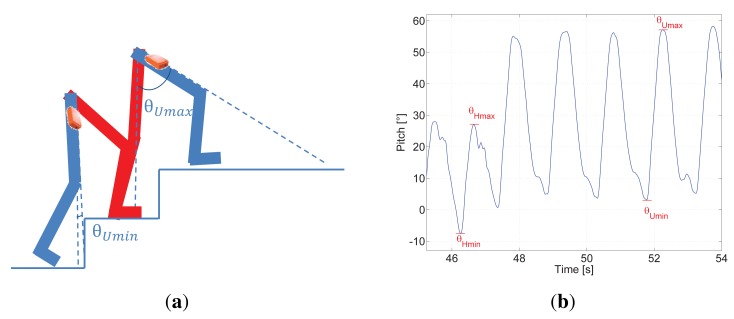
**(a)** represents the legs position on stairs; (**b**) shows eight steps, the first two steps the pedestrian walks on the landing zone of the staircase and the last five steps the pedestrian walks up stairs.

Figure 3a represents the movement of legs while walking up stairs and Figure 3b shows seven steps, the first four occurred going up stairs and the following three walking on the landing zone of the staircase.

It is noticeable that *θ*_Umax_ is almost doubled compared to *θ*_Hmax_. This is caused by the pedestrian raising his/her leg in order to reach the following step up of the staircase. In addition, |*θ*_Umin_| is slightly smaller than *θ*_Hmin_. It is convenient to use the absolute value because, depending on the height of the step of the staircase, way of walking of every pedestrian, his/her tiredness, hurry, *etc*. *θ*_Umin_ oscillates around 0 degrees, *i.e.*, it can be slightly positive or slightly negative.

Additionally, it is observable that the pitch amplitude by walking up stairs Δ*θ*_U_, which is computed as in Equation (4), is notably larger than Δ*θ*_H_ and the double maximum peak disappears appearing in contrast a double minimum peak.

**Figure 4:**
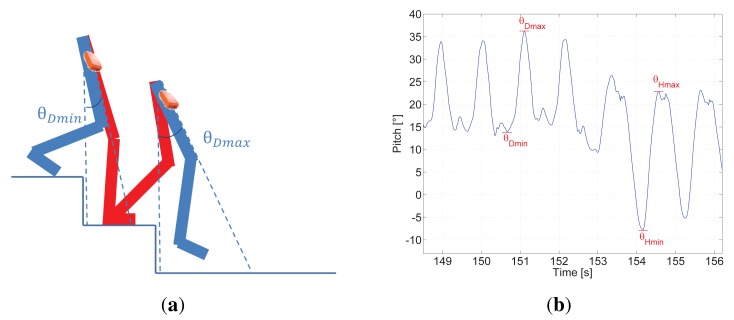
**(a)** represents the legs position on stairs; (**b**) shows seven steps, the first four steps the pedestrian walks down stairs and the last three steps the pedestrian walks on the landing zone of the staircase.

Figure 4a represents the movement of legs while walking down stairs and Figure 4b shows seven steps, the first four occurred going down stairs and the following three walking on the landing zone of the staircase.

It is noticeable that *θ*_Dmin_ has a positive value in contrast to *θ*_Hmin_. This is caused by the pedestrian moving his/her leg in order to reach the following step down of the staircase. In addition, *θ*_Dmax_ is slightly larger than *θ*_Hmax_.

Additionally it is observable that the pitch amplitude by walking down stairs Δ*θ*_D_, which is computed as in Equation (4), is notably smaller than Δ*θ*_H_ and the double maximum peak disappears appearing in contrast a double minimum peak.

Our step detector, as previously explained, consists of the identification of the maximum of the pitch angle because each maximum indicated as *θ*_Umax_ or *θ*_Dmax_ corresponds to a step. As shown, the pitch angle is adequate as well for detecting steps up and down in stairs.

### 4.3. Physical Activities Identification

This section presents the potential of the pitch angle to distinguish between different physical activities, particularly standing, walking, walking up stairs, walking down stairs and sitting.

**Figure 5:**
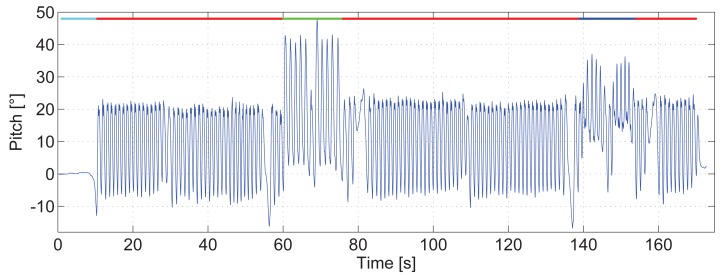
The blue line represents the estimation over time of the pitch angle, measured in degrees, during a multi-storey walk of a pedestrian with the sensor in the pocket.

Figure 5 shows the estimated pitch angle during a walk. In this walk, there are four clearly differentiated physical activities: standing is indicated by the cyan line; walking is indicated by the red line; walking up stairs is indicated by the green line; and lastly, walking down stairs is indicated by the blue line.

In this walk, the pedestrian started standing, and then, she walked through the corridor to the stairs. She walked up until the second floor of the building, and she walked until the end of the corridor and came back to the staircase. She descended again to the ground floor and walked to the starting point.

This figure shows how intuitive it is to detect activities by simply tracking changes in the pitch estimation. The algorithm for distinguishing walking on a horizontal surface, walking up stairs and walking down stairs proposed in this work is based on three parameters of the pitch angle estimation: *θ*_min_, *θ*_max_ and Δ*θ*.

The parameters *θ*_Hmin_, *θ*_Hmax_ and Δ*θ*_H_ are learned during the walk under the assumption that the pedestrian starts walking on a horizontal surface. Therefore, these values are personalized for an optimal performance and continuously adapted to the pedestrian's mood, hurriedness, tiredness, *etc*.

In order to determine if the pedestrian is walking up stairs, down stairs or horizontally, the parameters *θ*_min_, *θ*_max_ and Δ*θ* are stepwise extracted and compared to the reference parameters $\bar{\theta_{\text{Hmin}}}$, $\bar{\theta_{\text{Hmax}}}$ and $\bar{\Delta \theta_{\text{H}}}$, which are the result of averaging the last horizontal steps:
**if**
$\Delta \theta \gg \bar{\Delta \theta_{H}}\&\&\theta_{\max}\gg \bar{\theta_{\text{Hmax}}}$**then** *θ*_Umax_ = *θ*_max_; *θ*_Umin_ = *θ*_min_; Δ*θ*_U_ = Δ*θ*;**end****else if**
$\Delta \theta \ll \bar{\Delta \theta_{H}}\&\&\theta_{\min}\gg \bar{\theta_{\text{Hmin}}}$**then** *θ*_Dmax_ = *θ*_max_; *θ*_Dmin_ = *θ*_min_; Δ*θ*_D_ = Δ*θ*;**end****else** *θ*_Hmax_ = *θ*_max_; *θ*_Hmin_ = *θ*_min_; Δ*θ*_H_ = Δ*θ*;**end**

The thresholds needed in order to determine if the pedestrian is walking up stairs or down stairs can be universal or personalized. The more accurate option is to calibrate the system to correctly select them adapted to each pedestrian.

Regarding the standing and sitting activities, their main characteristic is the value of the pitch, which is almost constant. The pitch value lies around zero degrees when the pedestrian is standing and around 45-90 degrees if the pedestrian is sitting.

**Figure 6:**
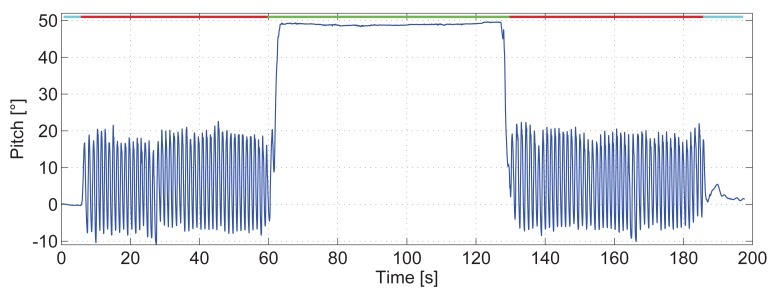
Pitch angle estimation of a walk, including standing, walking and sitting.

Figure 6 shows the pitch estimation of a pedestrian doing the following activities: standing, walking, sitting, walking and standing. Standing is indicated by the cyan line; walking is indicated by the red line and sitting is indicated by the green line.

The activities standing and sitting are only checked when no steps are detected. Then, the distinction between them only depends on the value of the pitch angle.

The identification of the physical activity distinguishes if the step taken was on a horizontal surface, was a step up or a step down. This distinction is of high interest for aiding the navigation, being the basis of the vertical displacement estimator that will be explained in Section 6. Furthermore, some applications, such as monitoring patients in hospitals or elderly care, usually need the fusion of the navigation and identification of physical activities.

## 5. Step Length Estimator

In [9], the step length estimator based on the pitch angle and the measurement campaign with 18 volunteers participating was already presented. Therefore, only the most important characteristics will be explained in this section for completeness. Additionally, an overview of the step length estimator if the pedestrian is running will be presented, and a calibration method for adapting the step length estimator model to each pedestrian will be proposed. All data presented in this section have been extracted from the aforementioned measurement campaign.

**Figure 7:**
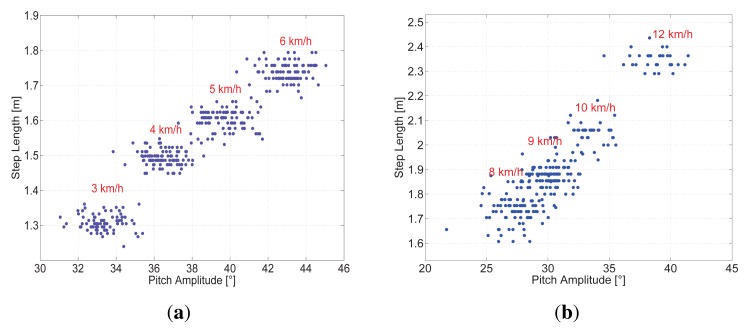
The points represent the steps from one volunteer. Each cloud of points represents a different speed. (**a**) when he was walking; (**b**) when he was running.

It was already assessed that a relationship between the pitch amplitude, Δ*θ*, and the step length, *S*, exists. Figure 7 shows the pitch amplitude in degrees against the step length in meters for one volunteer. In Figure 7a the pedestrian walks from 3 km/h until 6 km/h and Figure 7b shows the data of the pedestrian running from 8 km/h until 12 km/h.

Each cloud of points represents a different speed, and each point represents a step. This figure shows the clear trend of the data obtained. It indicates that, the larger the pitch angle is, the larger the step length will be. The figure also shows that the higher the pedestrian speed is, the larger the step length is and, consequently, the larger the pitch angle will be. This can be noticed because the steps recorded at each speed are sorted increasingly.

The shape of the pitch angle estimation is the same for walking and running as long as the surface is horizontal for both activities (see Figure 2b). Therefore, it was expected that, by increasing the speed, both the step length and pitch amplitude also increase. However, the steps of Figure 7a,b together do not lie in a single line, as expected. This is due to the running cycle, which has a stage when none of the feet touch the floor. The different gait cycle modifies the opening angle of the legs, thus the pitch amplitude.

In any case, Figure 7a,b show the same trend; therefore, the step length for running and walking can be modeled analogously. However, the running activity is out of the scope of this article; thus, from here on, it will not be taken into account.

The vast set of steps recorded at different walking speeds by 18 volunteers of different ages, genders, heights and weights yields the first linear step length model based on the pitch angle:

(5) $$S=a\cdot \Delta \theta_{\text{H}}+b$$

where *S* is the estimated step length measured in meters taking into account the pitch amplitude on horizontal surfaces, Δ*θ*_H_, in degrees. The constants *a* and *b* are the parameters fitting the regression line of each pedestrian.

### 5.1. Model Calibration

Fitting the data of the experiments with a regression line, as Equation (5) shows, is the simplest model assumption. Having the correct parameters *a* and *b* for each pedestrian is of crucial importance, but no calibration method has been proposed so far.

**Figure 8:**
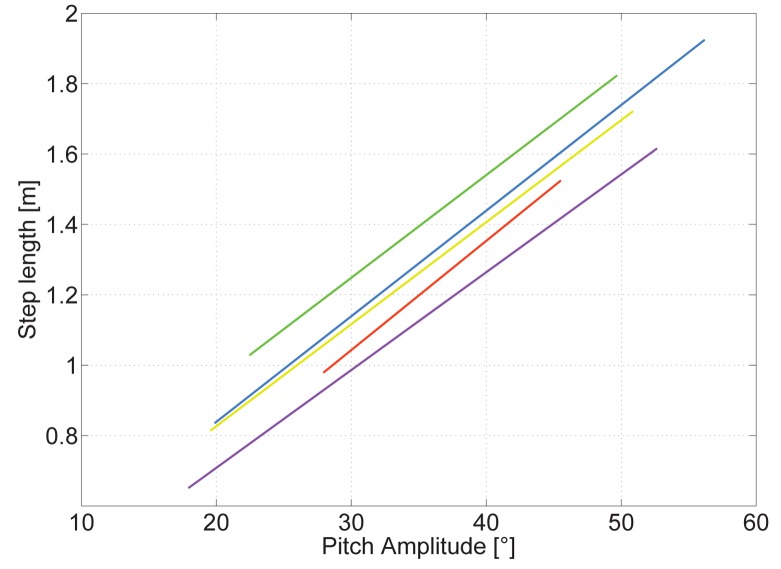
Regression lines of five volunteers walking at different speeds between 2.5 km/h and 6.5 km/h.

A closer investigation of the regression lines of different volunteers shows that the slope *a* is very similar among them. Figure 8 shows the regression lines in different colors of five volunteers who repeated the experiment without the running machine, walking directly on a horizontal surface.

The universal parameters are computed taking into account the complete set of steps of all volunteers. In Figure 8, the universal regression line overlaps the yellow line. This figure shows an overview of the error in the estimated step length for each volunteer if the universal parameters are used. The error has a range from almost 0 cm per step for the volunteers whose regression line is represented in yellow and blue to almost 14 cm per step for the volunteers whose regression lines are represented in green and violet.

From the experiments, it could not be concluded which pedestrian characteristics influence *a* and *b*. Biomechanical studies show that at any given walking speed, it is possible to select different combinations of step frequency and step length. However, individuals tend to constantly choose a specific step length for each walking speed [21]. Optimizing energy costs primarily determines the selection of a certain gait pattern.

A possible explanation of the similar slopes *a* of the regression lines is that all volunteers choose his/her step length at each speed basically regulated by the minimization of energy costs. This pattern is the same for all pedestrians.

There are, however, other characteristics, such as height, length of the legs, weight, gender, *etc.*, that cause differences between pedestrians. This is a possible explanation of the different offsets *b*.

Based on our results, we suggest a calibration method using the universal parameter *a*. Therefore, the pedestrian only has to walk a known distance at his/her preferred comfortable speed to find the personalized parameter *b*. This is a simple and quick calibration, which does not require additional infrastructure or additional user information, such as height or length of the leg.

## 6. Vertical Displacement Estimator

This section aims at explaining the novel vertical displacement estimator algorithm, which is based on the pitch angle. First, the experiments carried out in order to find a model will be detailed, and then, the model derivation will be explained.

In order to estimate the vertical displacement with the information of the pitch angle, a set of experiments has been carried out with the objective of finding a relationship between the pitch angle and the height of the steps of the staircase.

### 6.1. Experimental Setup

The experiment consists of recording the pitch information of steps taken walking up and down stairs. Almost 400 steps up and down have been recorded by one volunteer in order to study the relationship between pitch amplitude and the height of the steps of a staircase.

A wooden structure of adjustable steps, which varies between a height of 15 and 25 cm, has been built to carry out this experiment. The height of the steps has been chosen based on the document “Visual Interpretation of the International Residential Code” [22]. It specifies that the maximum height for the step of the staircase should be 19.7 cm, and for a spiral staircase, a maximum height of 24.1 cm is recommended. The minimum depth of a step of the staircase is 25.4 cm.

**Figure 9:**
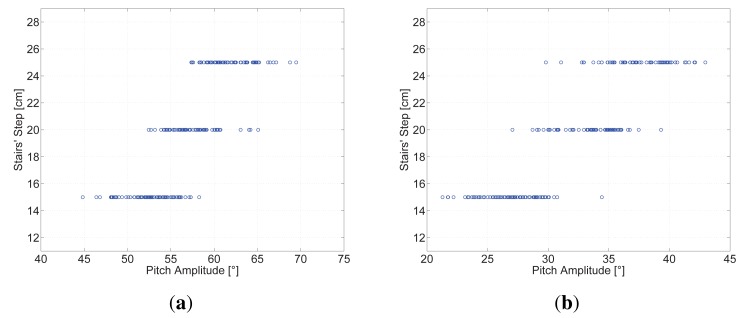
In this figure the points represent the steps recorded when the volunteer was walking up and down stairs on the adjustable wooden structure built for this experiment. (**a**) Steps recorded walking up stairs; (**b**) Steps recorded walking down stairs.

Figure 9 shows that a relationship between the pitch amplitude and the height of the steps of the staircase exists, indicating that the higher the step of the staircase is, the larger the pitch amplitude will be. The same behaviour is observed when the pedestrian walks up and down stairs.

The pitch angle has been measured using a precise fiber optic gyroscope (FOG), the DSP-1750 FOG from KVH. It has been mounted externally in the pocket with the help of a solid wood base, because the size of the FOG makes it impossible to introduce it in the pocket. The Allan variance analysis of a 10 h's turn rate recording reveals that the white noise of the fiber optic gyroscopes is two orders of magnitude smaller than the MTw gyroscopes' white noise. Additionally, the biases of the fiber optic gyroscopes can be considered stable within the length of the aforementioned measurements. Therefore, the pitch amplitude measured for this experiment will be considered the ground truth.

Figure 9 shows that a relationship between the pitch amplitude and the height of the steps of the staircase exists, indicating that the higher the step of the staircase is, the larger the pitch amplitude will be. The same behavior is observed when the pedestrian walks up and down stairs.

For assessing the results of the experiment of the wooden adjustable structure, the same has been done using the regular stairs of the DLR building office, whose height of the steps of the staircase is 17 cm.

Around 180 steps up and down have been recorded by the same volunteer walking on the five-floor staircase. The value of the estimated pitch amplitude for this particular height of steps is 53.07 ° and 26.62 ° for up and down stairs, respectively. These values are used to assess the experiments carried out with the wooden structure, whose results are shown in Figure 9.

### 6.2. Model Derivation

The simplest assumption for the behavior shown in Figure 9 is a linear model:

(6) $$V_{\text{U}}=e\cdot \Delta \theta_{\text{U}}+g$$

(7) $$V_{\text{D}}=h\cdot \Delta \theta_{\text{D}}+j$$

where *V*_U_ and *V*_D_ are the estimated vertical displacement measured in centimeters for up and down stairs, respectively. Δ*θ*_U_ and Δ*θ*_D_ are the pitch amplitude measured in degrees for steps up and down, respectively, and *e*, *g*, *h* and *j* are parameters, either personalized for each pedestrian or universal.

It is important to note that the pitch amplitude walking up and down stairs is due to the vertical and horizontal displacement of the pedestrian. If the stairs depth is larger than the standard, for example in parks outdoors, the model parameters should be re-calibrated, even for the same pedestrian.

The presented model is valid to estimate the height of the steps up and down of the vast majority of the staircases that can be found in every building, because it assumes a standard depth and focuses on the height of the step. Thus, the horizontal displacement is assumed. This is a reasonable assumption, because, as shown in [22], the staircases of any building are standardized and adapted to the length of the human foot. Additionally, the most important characteristic of the stairs is their height, which allows estimating the height between floors.

The algorithm gathers data when the pedestrian walks up and down stairs. There are small differences in the pitch amplitude, as shown in Figure 9. However, the vertical displacement is the same, because the height of the steps is the same for the whole staircase. The height of the steps of the staircase is estimated as the average of the pitch amplitude of all steps up and down using the models presented in Equations (6) and (7), respectively.

Even if the model of Equations (6) and (7) will be tested with different volunteers, an extension of the experiment described in Section 6.1 to more volunteers of different ages, genders and heights is desirable.

## 7. Experimental Results

This section gathers the most representative experiments that we have carried out in order to assess the algorithms presented before. For these experiments, the sensor was always introduced directly in the pocket without additional fastening.

### 7.1. Step Detector Assessment

To assess the performance of the proposed step detector, four different scenarios have been chosen. The scenario Horizontal 1 describes a rectangle of 100 × 80 m walking at a constant comfortable speed of 3.5 km/h. For the scenario Horizontal 2, the pedestrian walks the long rectangle side at a slow speed and the short side at a speed of 4.8 km/h. The goal of scenarios Upstairs and Downstairs is to test the detection of steps up and down in stairs, walking at a constant and comfortable speed. After 40 m of a straight line, the pedestrian enters the staircase and walks four floors up and then down. Finally, after leaving the staircase zone, the volunteer walks back to the starting point. The experiment log files and more information of these walks is available online [23].

The methods under study are the norm of the complete acceleration and its low-pass filtered version. The complete acceleration instead of the vertical acceleration is used to be robust to different sensor orientations. Regarding the pitch signal, both methods are studied, the zero-crossing detection (Z-C) and the method proposed in this article. The detection thresholds are adapted to the comfortable volunteer walking speed. Therefore, these parameters are constant during the experiments, thus not adapted to different scenarios. The results are shown in Table 1, where both the undetected steps (UD) and the false detections (FD) are detailed.

The comparison between the aforementioned algorithms is limited to the detection of steps, because it is not possible with the literature step detectors under study to distinguish between horizontal steps and steps up or down.

The experiments summarized in this table show that it is possible to successfully detect steps at a constant and comfortable speed, as shown in scenario Horizontal 1, with the four different algorithms under study.

We have applied a low-pass filter of 15 samples to the norm of the acceleration signal to detect steps with the second proposed method. The filter smoothing makes the signal contain less peaks that could yield false step detections. Therefore, it is recommended to filter the norm of the acceleration to avoid false detections. The smoothing helps in all scenarios.

**Figure 10:**
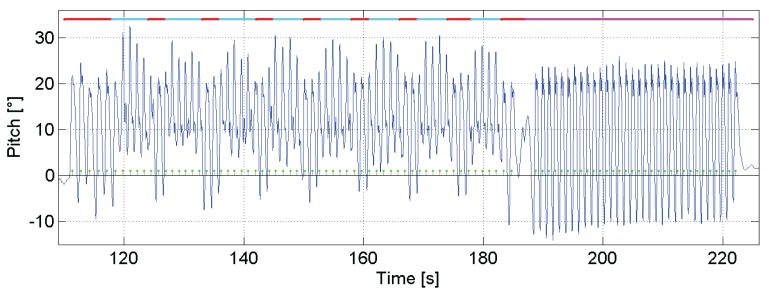
Pitch angle estimation of the scenario Downstairs. The green dots represent the truth of the taken steps. The upper colored line maps different zones of the walk: red for the landing zone of the staircase, cyan for the stairs and magenta for the corridor.

Figure 10 shows the pitch angle estimation for the scenario Downstairs. The green dots represent the truth of the taken steps. The pedestrian starts walking on the landing zone of the stairs of the upper floor. Then, the pedestrian takes the stairs to reach the ground floor. In the figure, the landing zones of the stairs are highlighted with a red line, and the stairs are highlighted with a cyan line. Finally, the pedestrian walks approximately 40 m in a straight line through the corridor. This is marked in the figure with a magenta line.

The zero-crossing-based pitch detector performs similarly to our algorithm; however, it is not able to detect all steps on the stairs, because zero-crossing does not occur, as shown in Figure 10 for the scenario Downstairs. The zero-crossing for walking up stairs, however, depends on the pedestrian, his/her mood, tiredness, *etc*. Therefore, the minimum of the pitch signal by walking up stairs can be slightly positive or slightly negative, as mentioned in Section 4.2. For the scenario Upstairs, the minimum pitch particularly for the volunteer has a slightly negative value; thus, the zero-crossing detects every step up.

As Figure 10 shows, the pedestrian walking speed is lower in the landing zones than in the corridor; therefore, the pitch amplitude is smaller, and the steps take more time. The vast majority of the steps taken in the landing zones are not detectable with the acceleration-based algorithms. The same problem appears in the scenario Horizontal 2, where the pedestrian was asked to walk the long rectangle side at a slow speed and the short side at a higher speed.

Pedestrians naturally adapt their walking speed and step length to the scenario or to their circumstances. Therefore, the changes of speed during the same walk represent a realistic scenario. In order to avoid losing steps in such situations, a possible solution consists of an adaptable threshold. Such an adaptable threshold, however, is not necessary with the detection based on the pitch angle. This is one of the greatest advantages of the pitch-based step detection.

Our step detector based on the pitch angle successfully detects every step of the presented scenarios and does not incur the false detections that are likely to happen, for example, when the pedestrian opens or closes doors, as Figure 10 shows for Second 185.

### 7.2. Step Length Estimator Assessment

In [9], an assessment of the proposed step length estimator based on the pitch angle was shown. Additionally, in this article, a comparison between our step length estimator and the well-known step length estimator based on the step frequency is analyzed.

**Figure 11:**
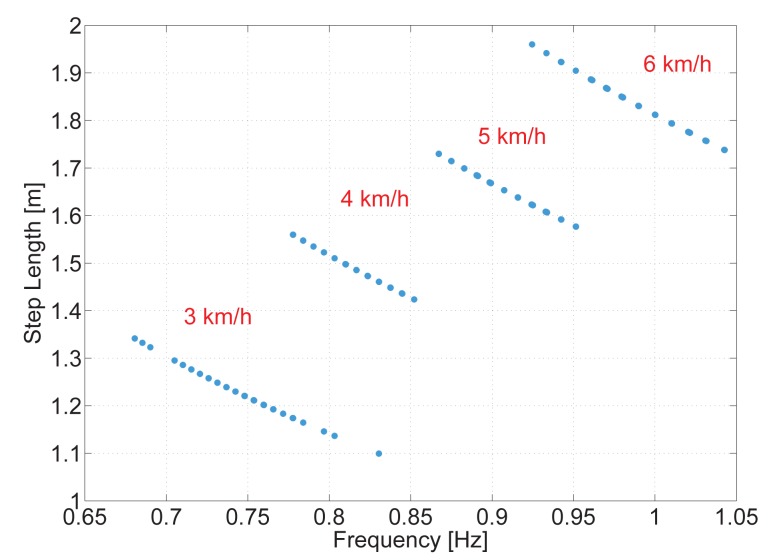
The points represent the steps from data gathered for one volunteer and each cloud of points represents a different speed.

To better and fairly compare the frequency step length model, its regression line has been extracted from the same data of the experiments with the running machine described in [9]. Figure 11 shows the steps from the same pedestrian shown in Figure 7a represented against the step frequency instead of the pitch amplitude. The shape of the clouds of points of each walking speed results from the restriction of the constant speed imposed by the running machine.

For this model, the following equation will be used:

(8) S=c⋅F+d

where *S* is the estimated step length measured in meters taking into account the step frequency (*F*) measured in Hertz. In order to fairly compare these methods, the constants *c* and *d* will be personalized parameters.

We have chosen to use the horizontal scenario described in Section 7.1, because the step length estimator based on the frequency does not cope with the vertical displacement of the pedestrian. The scenarios Horizontals 1 and 2 have been evaluated in this section. The scenario Horizontal 3 simulates a walk in a museum where the pedestrian walks slowly, at approximately 2 km/h. The step detector for this evaluation is the one proposed in this article, and it is used with both step length estimators.

Table 2 presents the errors of each method for the proposed scenarios. The parameters for both step length estimators are personalized for the volunteer and constant for all scenarios, thus not adapted to different waking speeds. The experiment log files and more information of these walks is available online [23].

The results in Table 2 for the scenario Horizontal 1 show that both methods perform similarly at a constant comfortable speed, 3.5 km/h. Such an accurate estimation of the length of the trajectory, with errors below 1%, is possible because the sensor is attached to the pedestrian's body. This is a clear advantage of the pocket location: the movements of the sensor are fully coupled with the movements of the pedestrian. This is, for example, not the case when the sensor is held in the hand.

The scenario Horizontal 2 shows less accurate results, which are caused by the change of walking speed. The speed changes are clearly the worst case for any step length estimator, because these algorithms are usually adapted to work optimally at a comfortable and quasi-constant walking speed.

The scenario Horizontal 3 represents an extreme situation for both step length estimators under study, because the walking speed is 2 km/h and the walk includes some stops. This speed is far from the optimal working zone of the regression line for both step length estimator models. The results show however higher error for the step length estimator based on the step frequency. A possible explanation is that the frequency was computed as the inverse of the time between steps. A more sophisticated approach uses the Fourier transform to find out the pedestrian walking frequency. However, this method requires periodicity, which is broken by the changes of speed by turning or stopping.

The above analyzed experiments show the benefits of using the proposed step length estimator when the sensor is in the pocket. The optimal working zone of the regression line of the pitch-based model is larger than the one based on the step frequency. Thus, the proposed model can cope with a wider range of walking speeds.

Additionally, it is interesting to study the results using a more complex model than a regression line. The representation of the step length against the pitch amplitude for a set of steps recorded at speeds, from 1 km/h to 10 km/h for one volunteer, is available online [23]. Such a wide range of speeds requires two different models, as mentioned in Section 5: walking and running.

### 7.3. Vertical Displacement Estimator Assessment

In order to assess the vertical displacement estimator, we have tested the inertial pocket navigation system with a multi-story walk in a real-world scenario.

**Figure 12:**
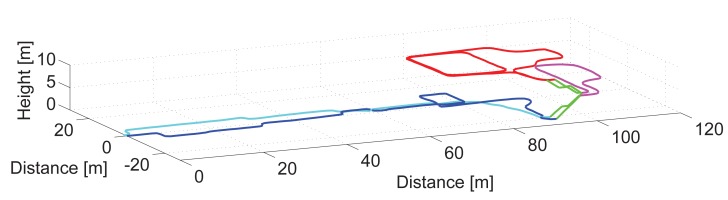
3D view of the odometry estimation of a 10-min's walk at the Deutsches Museum, Munich (Germany).

Figure 12 shows the 3D view of the estimated odometry of a 10 min's walk at the Deutsches Museum, which is a well-attended and well-known museum in Munich, Germany. The video, the experiment log files and more information is available online [23].

This odometry was estimated following the diagram of Figure 1, but using only a three-axis accelerometer and a three-axis gyroscope. We did not use the magnetometer in order to have unaided inertial positioning. No aid from other sensors, such as a barometer, WiFi access points, GNSSs, maps, *etc.*, was used.

The step detector used is the one proposed in Section 4, and the step length estimator used is the one proposed in Section 5 with the universal parameter *a* and the personalized parameter *b* for the volunteer. The drift in the heading has been compensated in order to evaluate only the influence in the odometry of the errors in the pitch angle that may cause errors in the step detector, step length estimator and vertical displacement estimator and the errors of the proposed algorithms themselves.

Based on the definition of a step (see Figure 2a), a step is counted between consecutive hits on the floor of the same foot. Therefore, the depth of each step of the stairs shown in Figure 12 has a length of 0.52 m, twice the standard depth of 0.26 m.

The main part of the trajectory is on the ground floor and is depicted in blue, dark blue for the initial part starting at (0,0,0) and light blue for the final part for walking back to the starting point. This area of the museum is filled with real ships and aircraft. The stairs are depicted in green.

First, the pedestrian visited the mezzanine, which is a narrow area depicted in magenta. The pedestrian went around it and took the stairs again to go up to the first floor, which is depicted in red. She walked around for a few minutes, repeating part of the trajectory, and finally, she again took the stairs to go back to the ground floor again. The way back to the main entrance, *i.e.*, the selected starting point of the walk, is depicted in light blue. On the way back, she did not choose to repeat the previous trajectory, but she surrounded two big ships, which are located between 10 and 50 m in the main exhibition hall.

**Figure 13:**
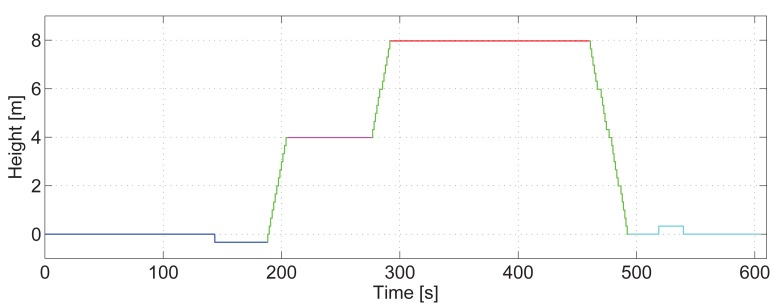
Height estimation of the walk shown in Figure 12 recorded at the Deutsches Museum, Munich (Germany).

Figure 13 shows the vertical displacement estimation of the pedestrian over time for the aforementioned walk. For clarity, the color code is the same as Figure 12.

This figure assesses that the vast majority of steps up and down are detected. However, the fact that the steps of the staircase are detected in pairs causes that, if a single step up or down is taken with the leg that does not have the sensor in the pocket, it may not be detected. This happens with the platform situated in the ground floor. The detector only detected the step down for Second 150, but not the step up. This platform was, however, correctly detected on the way back, as the light blue line represents. One extra step up is falsely detected in the first part of the staircase due to the same reason. The correct step up and down detection depends on the number of steps of the staircase (odd or even) and on the foot the pedestrian starts walking on for the stairs.

The estimation of the height of the steps of the staircase based on the amplitude of the pitch angle for up and down stairs is summarized in Table 3.

The error of *V*_U_ and *V*_D_ corresponding to the vertical displacement up stairs and down stairs, respectively, is below 1%. This implies an error of one meter every 100 m of vertical displacement of the pedestrian. We consider that the error is not acceptable if a confusion between consecutive floors is possible. Considering a separation of floors of three meters, an error of 1% in the vertical displacement implies that the pedestrian has to walk 300 m up or down in order to mistake one floor, and this is highly unlikely to happen.

Therefore, we conclude that it is possible and recommended to use the pitch angle, not only for detecting steps and estimating the step length, but also for estimating the height of the steps of the staircase and, consequently, the height between floors.

## 8. Conclusions

This article aims at extending the step-and-heading approach to 3D positioning. Algorithms for a pocket-mounted sensor are proposed, which do not require the use of additional sensors, such as barometers, or additional information, such as maps. This novel idea exploits the use of the pitch angle, information usually ignored in most of the navigation systems.

The proposed step detector offers several characteristics that outperform the state-of-the-art step detectors. First, the undetected and the falsely detected step statistics are better than the standard step detector based on the acceleration. Additionally, our detector does not require speed-dependent thresholds and detects also steps up and down in stairs. We also demonstrated that with the pitch angle, it is possible to identify five basic physical activities.

We compared our step length estimator based on the pitch angle in different scenarios with the well-known state-of-the-art step length estimator based on the step frequency. The results of the experiments show that our estimator is more robust for slow and high walking speeds and stops during the walk.

We presented a novel model that estimates the vertical displacement of the pedestrian based on the pitch angle. The results of our experiments show that, with the information of steps up and down in stairs, it is possible to use the proposed model to estimate the height of the stairs, thus solving 3D positioning with unaided inertial data.

## 9. Outlook

The presented algorithms based on the pitch angle are accurate and robust for real-world scenarios. However, this technique requires an accurate orientation estimation, because the position estimation is directly affected by the pitch and yaw angles. It is necessary to count with a refined orientation estimation algorithm in order to avoid errors.

The versatility of the pitch angle makes it possible to solve 3D positioning for the unaided inertial step-and-heading approach. Even if the stairs are the most common way to vertically move among different floors in buildings, lifts and mechanical stairs are not covered yet.

## Figures and Tables

**Table 1:** Performance comparison of different step detectors.

	**Horizontal 1**		**Horizontal 2**		**Upstairs**		**Downstairs**	

	**US[%]**	**FD[%]**	**US[%]**	**FD[%]**	**US[%]**	**FD[%]**	**US[%]**	**FD[%]**
∥Acc∥	0.4	5	63	12	33	2.3	33	12
LPF ∥Acc∥	0	0	65	3.6	36	0	23	0
Pitch (Z-C)	0	0	0	0	0	0	38	1
Pitch	0	0	0	0	0	0	0	0

**Table 2:** Performance comparison of different step length estimators.

	**Horizontal 1 [%]**	**Horizontal 2 [%]**	**Horizontal 3 [%]**
Frequency	0.19	6.25	9.63
Pitch	0.18	3.24	1.15

**Table 3:** Deutsches museum step height estimation.

	**Estimated Height [cm]**	**Real Height [cm]**	**Error [%]**
*V*_U_	33.1	33.2	0.3
*V*_D_	32.9		0.9
